# Dataset of *Schinus terebinthifolius* essential oil microencapsulated by spray-drying

**DOI:** 10.1016/j.dib.2023.108927

**Published:** 2023-01-25

**Authors:** Regina da Silva Acácio, Aracelis Jose Pamphile-Adrian, Pedro Pablo Florez-Rodriguez, Johnnatan Duarte de Freitas, Henrique Fonseca Goulart, Antônio Euzébio Goulart Santana

**Affiliations:** a Instituto de Química e Biotecnologia, Universidade Federal de Alagoas, Av. Lourival Melo Mota, s/n, Tabuleiro do Martins, 57072-970, Maceió, AL, Brazil; b Campus de Engenharias e Ciências Agrárias, Universidade Federal de Alagoas, 57100-000, Rio Largo, AL, Brazil; c Instituto Federal de Alagoas, R. Mizael Domingues, 530, Centro, 57020-600, Maceió, Alagoas, Brazil

**Keywords:** Microencapsulation, Repellant activity, Maltodextrin, Arabic gum

## Abstract

Schinus terebinthifolius Raddi has been extensively studied due to its antioxidant, anti-inflammatory and antibiotic properties. Recently, its seeds have been tested against some insect pests as an insecticide, repellent and antifungal agent. Microencapsulation by spray-drying is widely used in the food and drug industries, as well as in the microencapsulation of essential oils, since it protects the oils against several effects, such as oxidation and thermal degradation, thus optimising its use. The aim was to microencapsulate *S. terebinthifolius* essential oil by spray-drying maltodextrin and arabic gum as encapsulating agents and SiO_2_ as a colloidal adjuvant. The morphology of the microcapsules was analysed by scanning electron microscopy (SEM), which evidenced mainly regular spherical-shaped particles with sizes between 5 and 10 µm. The thermal stability was studied by thermogravimetric analysis-differential scanning calorimetry (TGA-DSC), and the microcapsules were stable at temperatures up to 200°C. The microencapsulating agents and the spray-drying technique produced microcapsules capable of protecting the essential oil against external effects, such as thermal degradation.

**Specifications Table**

**Table:** 

Subject	Chemistry
Specific subject area	Chemical Engineering: Process Chemistry and Technology
Type of data	Table; Figure
How the data were acquired	The essential oil of *Schinus terebentifolius* was obtained by hydrodistillation using a modified Clevenger-type apparatus. The essential oil, as a 10 ppm solution in double distilled HPLC grade hexane (Merck, Darmstadt, Germany), was analysed by gas chromatography. The microcapsules were obtained by a spray-drying technique in a Mini Spray Dryer 1.0 LabMaq, Brazil. The chemical characterisation of the microencapsulated oil was performed by gas chromatography using a GC-2010 Plus (Shimadzu, Tokyo, Japan). The morphological characterisation of the microcapsules was conducted in two different ways. First, the encapsulated product was metallised in a QUORUM Q150R ES with a current of 45 mA for 200 s. Second, elemental analysis was performed by X-ray energy dispersive spectroscopy (EDS) using an SDD detector. The thermal degradation of the samples was performed in SDT650 equipment (TA Instruments).
Data format	Raw and analysed.
Description of data collection	To prepare the emulsions, the micro-encapsulation materials (maltodextrin, arabic gum and SiO_2_) were dissolved in a water/ethanol solution at 25°C, and this mixture was stirred until complete dissolution. Atomisation drying was performed with a controlled airflow and air pressure, and with clockwise rotation. SEM analysis was performed. Samples were subjected to a heating ramp from 35 to 650°C (10°C/min) for thermal analysis.
Data source location	*Institution: Federal University of Alagoas* *City/Town/Region: Maceió - Alagoas* *Country: Brazil* *Latitude and longitude:* 9° 33′ 11.08″ S, 35° 46′ 30.02″ W; 9° 33′ 10.63″ S, 35° 46′ 30.22″ W
Data accessibility	Repository name: Mendeley Data Data identification number: DOI:10.17632/nz4g5kz766.1 Direct URL to data: https://data.mendeley.com/datasets/nz4g5kz766

## Value of the Data

• The seeds of *Schinus terebinthifolius* present activities in different areas, such as health, with antioxidant, anti-inflammatory, antibiotic and anticancer action [1], in the food area for their antimicrobial, antibacterial and antifungal activity [2,3], and in the agronomic area for their repellent and insecticidal action against insect pests [4].
• The data provide a microencapsulated spray-drying formulation of the essential oil of *S. terebinthifolius* that protects the oil against external effects, such as temperature and oxidation, and presents controlled release of the essential oil [5].
• The data included in the article provide a broad characterization of the physicochemical properties of the microcapsules of essential oil of S. terebinthifolius, including the chemical composition of the essential oil, the morphology, and the thermal behaviour of the microcapsules.
• The data provide the formulation of essential oil of *S. terebinthifolius* microcapsules, supporting research work in the areas of food technology, pharmaceuticals and agronomy, bringing benefits to the entire population.
• Using the spray-drying technique to prepare the formulation allows the microencapsulation methodology to be scaled up using biodegradable and non-toxic agents. All food, pharmaceutical and agronomy professionals can use the material.

## 1 Objective

The aim was to microencapsulate *S. terebinthifolius* essential oil by spray-drying maltodextrin and arabic gum as encapsulating agents and SiO2 as a colloidal adjuvant.

Essential oils are characterized as complex mixtures of low molecular weight compounds, being highly volatile and responsible for flavors, aromas, and different biological activities such as antimicrobial, antifungal, insecticidal, and others [6]. The essential oil of *S. terebinthifolius* is cited in the literature for its antioxidant, anti-inflammatory and antibiotic properties, however its use is limited due to its volatility and rapid degradation under the influence of light, heat, and an oxidizing atmosphere [1]. The microencapsulation technique has been explored in sectors such as the food and pharmaceutical industries and is proposed for use in agriculture because it provides protection to active ingredients against external effects such as oxidation and degradation, in addition to providing controlled release of compounds [7].

## 2 Data Description

The essential oil of *Schinus terebinthifolius* Raddi fruits was obtained from hydrodistillation in a modified Clevenger-type apparatus. Table 1 presents the chemical composition of the essential oil by gas chromatography/mass spectrometry using a GCMS-QP2010 Ultra (Shimadzu, Tokyo, Japan). Fourteen compounds were identified in the essential oil of *S. terebinthifolius* fruits, corresponding to 96.13% of the total oil.

**Table 1:** Chemical composition of the essential oil of *Schinus terebinthifolius* Raddi fruits.

Compound	RT (min)	KI	*Peak Area %
4-Aliloxy-2-methyl-2-pentanol	6.870	899	0.06
Tricyclene	7.493	915	0.73
α-Tujene	7.620	918	0.08
α-Pinene	8.012	927	33.49
Canfene	8.503	940	0.66
β-Pinene	9.593	966	49.08
Mircene	10.213	982	0.72
3-Carene	11.183	1004	5.85
β-Phelandrene	11.564	1011	0.62
Bornilene	11.929	1018	1.21
α-Terpineol	20.121	1158	0.71
Bornil acetate	27.108	1266	0.73
Citronelil acetate	31.270	1329	0.10
Germacrene D	38.998	1466	2.08
Total identified	-	-	96.13

RT = retention time for GC-FID; KI = linear retention time for GC-FID calculated by Eq. (3).

⁎ Calculated by Eq. (2). Average value of three chromatograms.

**Fig. 1:**
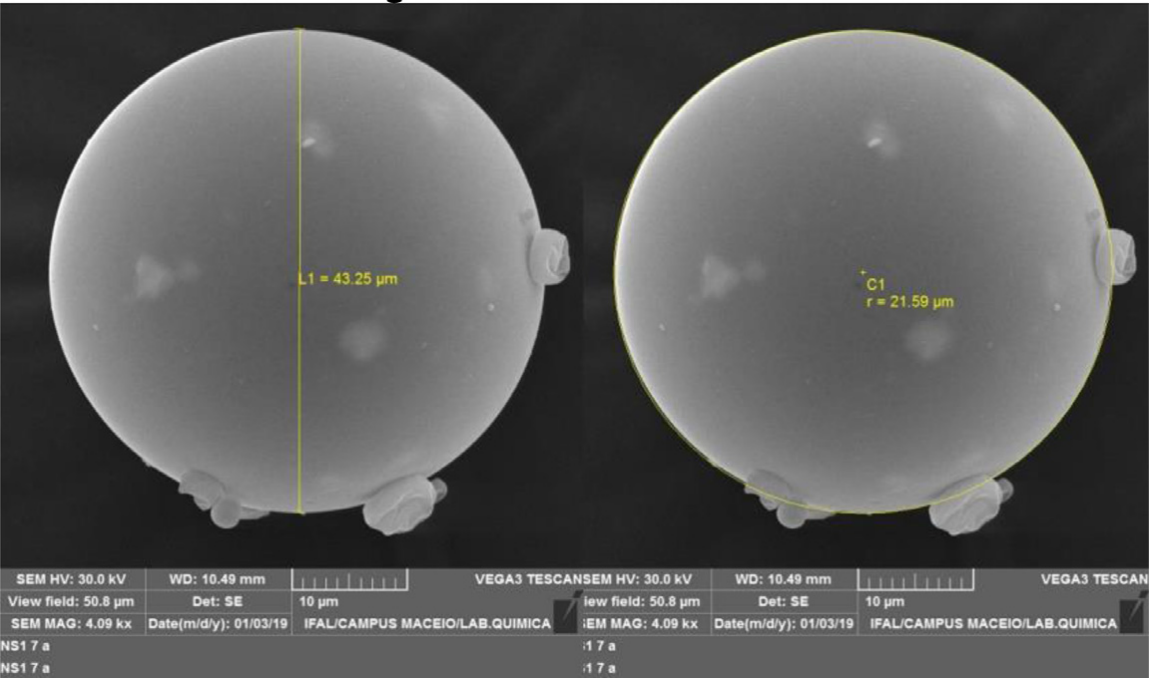
SEM images of an isolated metallised microcapsule, MD:AG:SiO_2_:EO, where MD is maltodextrin, AG is arabic gum, SiO_2_ is silicon dioxide, and EO is essential oil.

The morphology of the microcapsules was analysed by scanning electron microscopy (SEM). Fig. 1 shows images of the metallised MD:AG:SiO_2_:EO sample, where MD is maltodextrin, AG is arabic gum, SiO_2_ is silicon dioxide, and EO is essential oil. The microcapsules presented a circular shape and an apparent absence of pores. The evident lack of fissures in the walls or porosity in the surface of the particles indicates complete coverage of the nucleus by the wall materials.

**Fig. 2:**
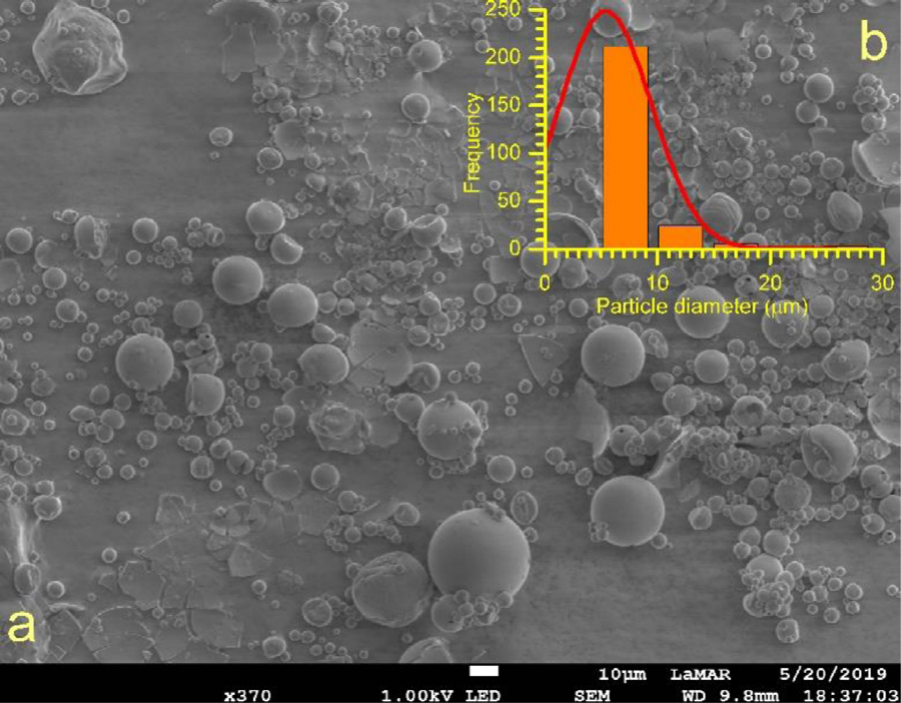
SEM image of the MD:AG:SiO_2_:EO microcapsule obtained without metallisation (A) and the particle size distribution (B).

Fig. 2 shows an SEM image of the MD:AG:SiO_2_:EO microcapsule obtained without metallisation (A) and the particle size distribution (B). The particle size distribution shows that 84% of the microcapsules present diameters between 5 and 10 µm.

**Fig. 3:**
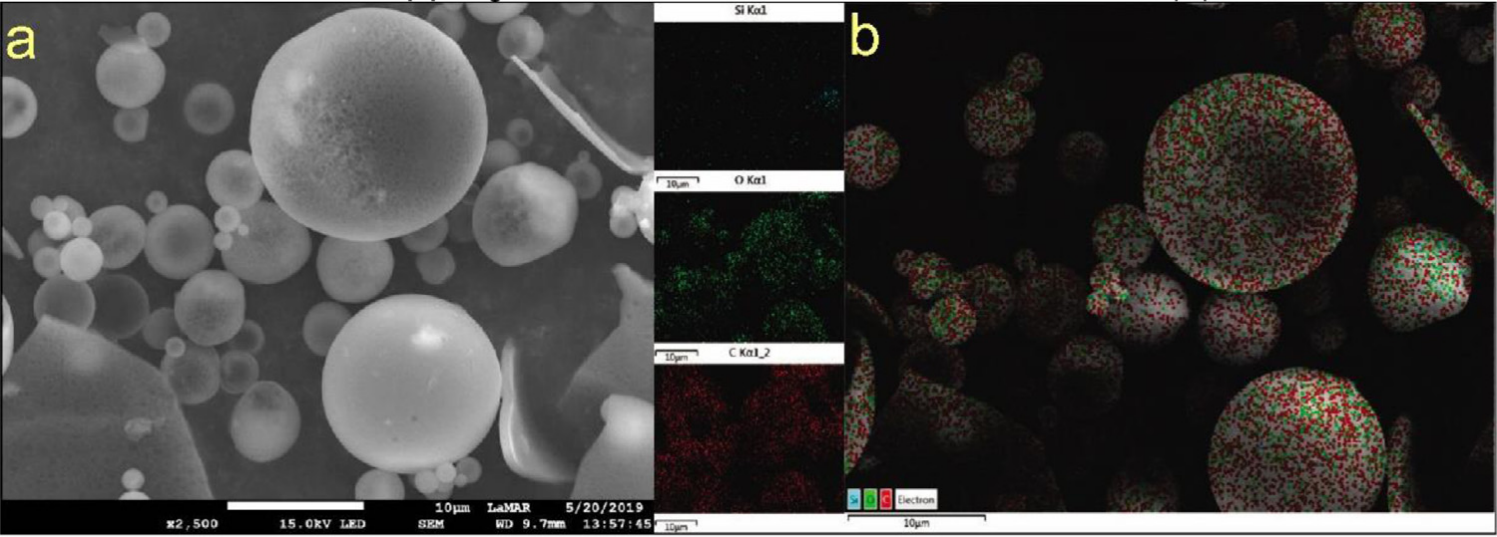
SEM image of the MD:AG:SiO_2_:EO microcapsule without metallisation (a); EDS mapping of C, O and Si for MD:AG:SiO_2_:EO (b).

Scanning Electron Microscopy-Energy Dispersive Spectroscopy (SEM-EDS) analyses of the MD:AG:SiO_2_:EO microcapsule without metallisation were performed to investigate whether the SiO_2_ is only present in the formulation as a dispersing agent, that is, to know whether the microparticles were formed by silicon or by carbon from the carbohydrates (maltodextrin and arabic gum). Fig. 3 shows an SEM image of the MD:AG:SiO_2_:EO microcapsule without metallisation (a); EDS mapping of C, O and Si for the MD:AG:SiO_2_:EO sample proves that the microcapsules are made of carbon and oxygen (b).

**Fig. 4:**
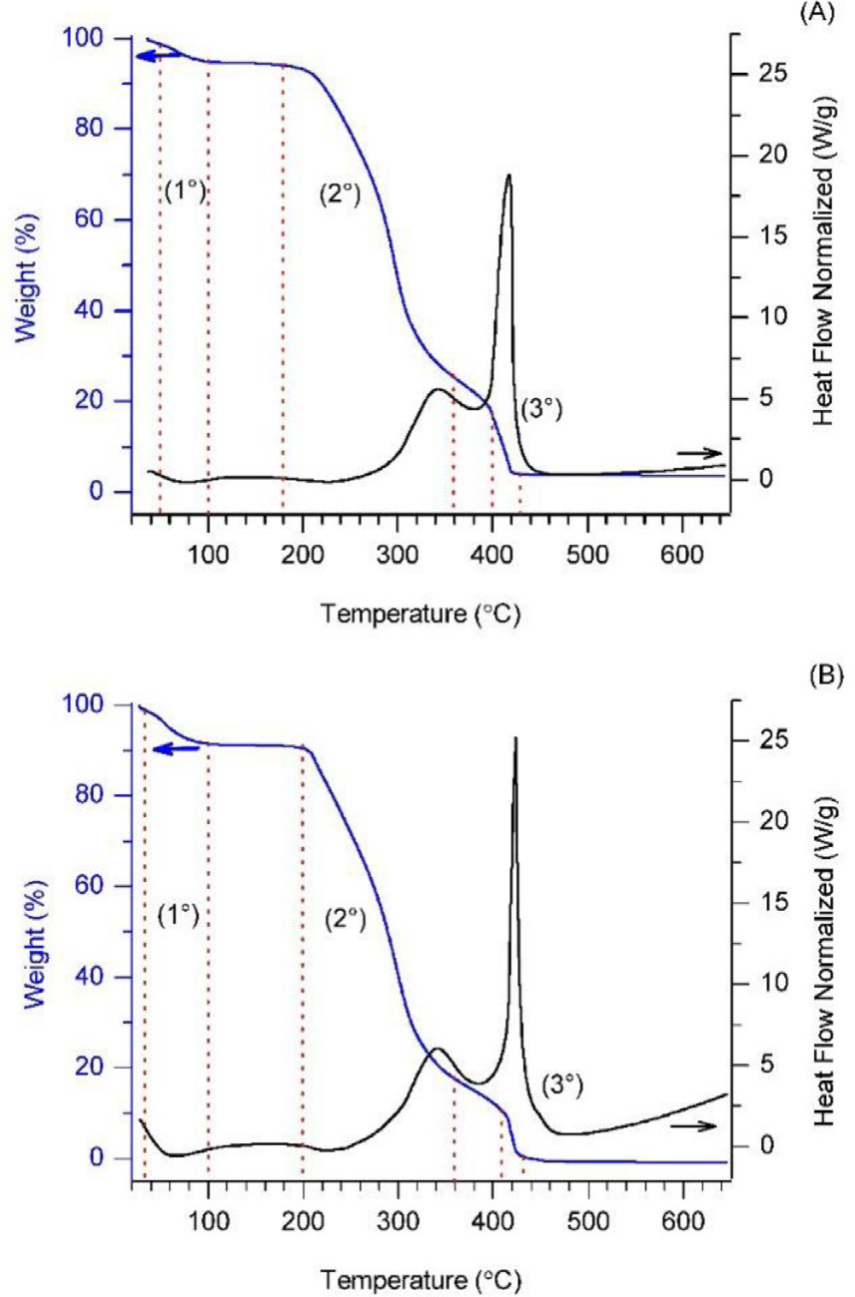
TGA profiles for MD:AG:SiO_2_ (A) and MD:AG:SiO_2_:EO (B).

Thermogravimetric analysis profiles for MD:AG:SiO_2_ and MD:AG:SiO_2_:EO are shown in Fig. 4. Both profiles presented three weight losses. The first one, between 49 and 100°C, represents 4.9% and 7.2% of the total mass for MD:AG:SiO_2_ and MD:AG:SiO_2_:EO, respectively, and is attributed to adsorbed water. The second weight loss occurred between 190 and 360°C for MD:GA:SiO_2_ and between 200 and 370°C for MD:AG:SiO_2_:EO, representing 68.2% and 74.3% of the total mass. These losses are attributed to the degradation of the organic wall materials (maltodextrin, arabic gum and essential oil), with only the SiO_2_ remaining, which indicates that they are stable at temperatures up to 190°C. The third weight loss occurred between 380 and 425 °C for MD:AG:SiO_2_ and between 410 and 430°C for MD:AG:SiO_2_:EO, representing 18.0% and 9.8% of the total mass, respectively. The raw and analysed data are available as supplementary documents (https://data.mendeley.com/datasets/nz4g5kz766/2) [6].

## 3 Experimental Design, Materials and Methods

### 3.1 Obtaining pink pepper tree (*S. terebinthifolius*) essential oil

The fruits of the pink pepper tree (*S. terebinthifolius*) were collected in the A. C Simões Campus of Alagoas Federal University, Brazil (9° 33’ 11.08’’ S, 35° 46’ 30.02’’ W; 9° 33’ 10.63’’ S, 35° 46’ 30.22’’ W) in October 2017. The vegetal material was identified by Rosângela P. Lyra Lemos of the MAC herbarium of the Environment Institute of Alagoas (IMA-AL), where a botanical exsiccate was deposited with register number 63595. The EOs were extracted from the fruits by hydrodistillation, according to the method used by the AOAC [7], using a modified Clevenger-type apparatus coupled to a 12 L volumetric flask. The dry fruits were crushed and submitted to steam distillation (1000 g in 7 L of distilled water) for 4 h. After separation of the aqueous phase, the oil portion was stored in amber glass and refrigerated (- 4 °C) until required.

The extraction was performed in quadruplicate, and the oil yield was calculated by the mass ratio of obtained oil and crushed vegetal material (Eq. (1)).

(1) $$\text{Yield}=\frac{mass\;of\;obtained\;oil}{mass\;of\;crushed\;vegetal\;material}\times 100$$

### 3.2 Chemical characterisation of the pink pepper essential oil by gas chromatography

The samples were analysed by gas chromatography using a GC-2010 Plus (Shimadzu, Tokyo, Japan) equipped with a DB-5 capillary column (30 m x 0.25 mm x 0.25 µm Agilent Technologies, USA) and FID detector. The samples were diluted with double-distilled HPLC grade hexane (Merck, Darmstadt, Germany) to 10 ppm. The samples (1 µL) were injected in splitless mode. The carrier gas was H_2_ with a pressure of 77.6 kPa, the injector temperature was 260°C, and the detector temperature was 360°C. The temperature program was initiated at 60°C, kept for 3 min, then increased to 300°C (8°C/min) and held for 10 min.

The proportion between compounds was calculated by the peak area percentage (Eq. (2)). The compounds were identified through the comparison of their retention times (RTs), and their linear retention indexes (KI) were determined by injecting a standard solution of n-alkanes with carbon numbers in the range C7-C30 and calculated using Eq. (3).

(2) $$Peak\;Area\;\%=\frac{Peak\;Area_{i}}{\sum_{i}Peak\;Areas}\times \;100$$

where i = compound of interest

(3) $$KI=\left[n+\frac{\left(T_{u}-T_{n}\right)}{\left(T_{N}-T_{n}\right)}\right]\times \;100$$

where:

KI = Kovats retention index
n = number of carbons in the alkane preceding the compound of interest
N = number of carbons in the alkane following the compound of interest
T_u_ = retention time of the unknown compound
T_n_ = retention time of the preceding alkane
T_N_ = retention time of the following alkane

The qualitative analysis of the compounds of the selected oils was performed in a GCMS-QP2010 Ultra (Shimadzu, Tokyo, Japan) equipped with a DB-5 capillary column (30 m x 0.25 mm x 0.25 µm i.d. Agilent Technologies, USA) and a quadrupole mass spectrometer. The samples (1 µL) were injected with a split ratio of 1:3 and an injector temperature of 260°C. The carrier gas was He with a pressure of 72.8 kPa. The temperature program used was the same program used for the GC-FID analysis. The ionisation was conducted by electron impact with an ionisation voltage of 70 eV, the ion source temperature was 290°C, and the detection was performed in scan mode from 35 to 400 m/z. The mass spectra were compared to those available in the commercial libraries NIST08, NIST08s and Wiley 275 L and those corresponding to synthetic patterns.

### 3.3 Microencapsulation of pink pepper tree essential oil

Maltodextrin DE-20 (Advanced Nutrition), arabic gum (LabSynth) and Aerosil® 200 (colloidal silicon dioxide – LabSynth) were used as encapsulating agents. A solution of 20% ethanol in MiliQ® ultrapure water was used as a solubilising agent.

To prepare the emulsions, the wall materials were dissolved in the water/ethanol solution at 25°C, and this mixture was agitated until complete dissolution. The total concentration was set at 30%, of which 70% was maltodextrin, 25% arabic gum and 5% silicium dioxide. The pink pepper essential oil was added to the hydrated wall material at a concentration of 20% relative to the total solids. An emulsion was formed under magnetic stirring at 1000 rpm (magnetic stirrer IPAS, model IKA® C-MAG HS 7).

Atomisation drying was performed in a spray dryer (LabMaq, model MSDi 1.0; Ribeirão Preto, Brazil) with a dual fluid atomising nozzle of 1.0 mm diameter, air flow of 30 L/min, air pressure of 3.0 bar and an airflow of the dryer of 3040 L/min. The dryer was fed at a flow of 0.32 L/h, with clockwise rotation. The air inlet temperature was 140 ± 2 °C and the outlet temperature was 104 ± 3 °C.

The prepared sample was named MD:AG:SiO_2_:EO, referring to the maltodextrin – arabic gum – silicium dioxide – pink pepper essential oil composition. A second sample was prepared using only the encapsulating agents and named MD:AG:SiO_2_ (maltodextrin – arabic gum – silicium dioxide).

### 3.4 Morphological characterisation by scanning electron microscopy (SEM) and by Energy Dispersive Spectroscopy (EDS)

For the morphological characterisation, the microcapsules were metallised in a QUORUM Q150R ES with a current of 45 mA for 200 s. The SEM images of the metallised samples were obtained at the Microscopy Laboratory of the Alagoas Federal Institute (Maceió, Brazil) in a TESCAN VEGA3, working in accelerating voltages between 5 and 30 kV in different magnifications.

To confirm the walls of the microcapsule, SEM images were also obtained without metallisation in a JEOL JSM 7100F microscope equipped with an emission field electron source, with an accelerating voltage between 1 and 15 kV in different magnifications. Additionally, elemental analysis was performed by X-ray energy dispersive spectroscopy (EDS) using a solid state detector (SSD).

The average particle diameter was measured in 250 microcapsules in PixelPro software.

### 3.5 Thermogravimetric analysis-differential scanning calorimetry (TGA-DSC)

The thermal degradation of the samples was performed at the laboratory of the Catalysis and Chemical Reactivity Group of the Alagoas Federal University (Maceió, Brazil) using SDT650 equipment (TA Instruments). The MD:AG:SiO_2_ and MD:AG:SiO_2_:EO samples (7.809 mg and 3.834 mg, respectively) were submitted to a heating ramp from 35 to 650°C (10°C/min) using synthetic air with a flow of 50 mL/min.

## Ethics Statements

None.

## CRediT authorship contribution statement

**Regina da Silva Acácio:** Conceptualization, Methodology, Writing – original draft. **Aracelis Jose Pamphile-Adrian:** Methodology, Data curation, Writing – original draft. **Pedro Pablo Florez-Rodriguez:** Investigation, Methodology, Resources, Supervision. **Johnnatan Duarte de Freitas:** Methodology, Investigation. **Henrique Fonseca Goulart:** Conceptualization, Writing – review & editing, Project administration. **Antônio Euzébio Goulart Santana:** .

## Declaration of competing interest

The authors declare that they have no known competing financial interests or personal relationships that could have appeared to influence the work reported in this paper.

## Data Availability

Data on spray-drying microencapsulation of Schinus terebinthifolius essential oil (Original data) (Mendeley Data).

## Appendix Supplementary materials

Image, application 1

## References

[1] Bendaoud H., Romdhane M., Souchard J.P., Cazaux S., Bouajila J. (2010). Chemical composition and anticancer and antioxidant activities of schinus Molle L. and Schinus Terebinthifolius Raddi berries essential oils. J. Food Sci..

[2] Da Silva Dannenberg G., Funck G.D., Mattei F.J., Da Silva W.P., Fiorentini Â.M. (2016). Antimicrobial and antioxidant activity of essential oil from pink pepper tree (Schinus terebinthifolius Raddi) in vitro and in cheese experimentally contaminated with Listeria monocytogenes. Innov. Food Sci. Emerg. Technol..

[3] da S. Dannenberg G., Funck G.D., da Silva W.P., Fiorentini Â.M. (2019). Essential oil from pink pepper (Schinus terebinthifolius Raddi): Chemical composition, antibacterial activity and mechanism of action. Food Control.

[4] Silva P.R.C., Camaroti J.R.S.L., Almeida W.A., Ferreira E.C.B., Paiva P.M.G., Barros R., Napoleão T.H., Pontual E.V. (2019). Schinus terebinthifolia leaf extract is a larvicidal, pupicidal, and oviposition deterring agent against Plutella xylostella. South African J. Bot..

[5] Nguyen T.T.T., Le T.V.A., Dang N.N., Nguyen D.C., Nguyen P.T.N., Tran T.T., Nguyen Q.V., Bach L.G., Thuy Nguyen Pham D. (2021). Microencapsulation of essential oils by spray-drying and influencing factors. J. Food Qual..

[6] R. Acácio, A.E.G. Santana, Data on spray-drying microencapsulation of Schinus terebinthifolius essential oil, 2 (2022). doi:10.17632/NZ4G5KZ766.2.

[7] Moran J.W., Turner J.M., Coleman M.R. (1995). Determination of monensin in edible bovine tissues and milk by liquid chromatography. J. AOAC Int..

